# Principles to Guide the Development of Population Health Incentives

**Published:** 2010-08-15

**Authors:** Robert H. Haveman

**Affiliations:** University of Wisconsin-Madison, Department of Economics and La Follette School of Public Affairs

## Abstract

Improving population health is not simple. Many instruments are available for changing behavior and consequent outcomes. However, the following basic principles should guide development of any incentive arrangement: 1) identify the desired outcome, 2) identify the behavior change that will lead to this outcome, 3) determine the potential effectiveness of the incentive in achieving the behavior change, 4) link a financial incentive directly to this outcome or behavior, 5) identify the possible adverse effects of the incentive, and 6) evaluate and report changes in the behavior or outcome in response to the incentive.

A wide range of financial and nonfinancial incentives is available to encourage efficient behaviors and discourage costly and unproductive ones. Evidence for the beneficial effects of incentive programs has been slow to emerge, partly because such evidence must show how behaviors have changed because of the incentive. Nevertheless, the potential for incentive programs in health care seems large, and research should support their design and assess their effect.

## Premise of Performance Incentives

Microeconomics is the study of how individuals, households, and businesses decide to allocate resources. These decisions are typically associated with decision makers who are closely tied to markets where goods or services are being bought and sold. However, similar allocation decisions are made in large organizations that are not directly connected to markets, such as government agencies, universities, public utilities, hospitals, and schools. The effect of these decisions on the output, quality, and cost of goods and services is used to judge the performance of the organization producing the good or service and of the members of the organization whose decisions contribute to production.

As of 2005, 75% of all private US companies based some part of employee pay on measures of performance determined by market signals, according to the Institute for Corporate Productivity (1). Managers of organizations that are not tightly connected to competitive market pressures must use different performance indicators to induce efficient and productive choices from their employees.

Deciding on performance incentives is not simple because many instruments are available for changing behavior and consequent outcomes. Some of these instruments are straightforward mandates that are imposed on decision makers; others involve financial penalties or rewards based on stated thresholds. Organized communication and consultation among employees, or "governance by committee," is another way to induce desirable performance.

The following are examples of incentive plans that have been adopted by private and public organizations:

- To promote a productive and trained state work force, a South Carolina program provides scholarship support to college students who maintain normal progress (2).
- In New York City, a pilot program pays parents to be involved in their children's school performance and health behaviors (3).
- One of the largest labor market policies in the United States, the Earned Income Tax Credit, is a cash incentive for increased earnings targeted at low-income workers.
- Medicare encourages physician training by providing financial subsidies to teaching hospitals to defray training costs (eg, salaries of medical residents and faculty).
- In health care, "pay for performance" is designed to improve efficiency and quality and to lower costs. Under these arrangements, health care providers are compensated for meeting performance measures.

## Basic Principles of Effective Incentives

The following basic principles may help clarify which financial and nonfinancial arrangements are appropriate for improving population health outcomes:

**Identify the desired outcome.** Although obvious, this straightforward principle is often violated. Consider, for example, a payment scheme designed to improve dermatologic screening for patients who are clinically determined to be at high risk of skin disorders. Incentives to reward primary care doctors for referring such patients to a dermatologist should be tied to the actual screening, not to the referral alone. Rewarding the actions of providers or patients for whom change is sought is the key to effective compliance (4).

**Identify the behavior change that will lead to this outcome.** In designing financial incentives, the desired action should be clearly identified. In the dermatologic screening example, the primary care provider must identify patients at risk, prescribe the activity, and take steps to ensure that the activity takes place.

**Determine the potential effectiveness of the incentive in achieving the behavior change**. The degree of provider or patient responsiveness to any financial incentive may vary widely. Understanding this response involves determining the extent to which the behavior targeted is amenable to change through the incentive. The size of the financial incentive should be appropriate to the effort required. If the perceived benefit of the action is exceeded by its perceived cost, the incentive will be ineffective. Another consideration in evaluating the proposed financial incentive is the importance of monetary gain for decision makers. A financial incentive will typically generate less response among wealthy decision makers than among lower-income decision makers.

**Link a financial incentive directly to this outcome or the behavior.** In the example of improving dermatologic screening, any financial payment should be directly tied to either the final outcome — documented examinations for high-risk patients — or to the actions of the primary care provider, for example, 1) identifying high-risk patients, 2) prescribing a dermatologic examination for them, 3) following up with patients to encourage the examination, and 4) documenting the results of the examination. In 1 option, a flat payment could be attached to each step in this process. Alternatively, payments could be graduated so that the payment for each step of the process would be higher than for previous steps. A graduated payment arrangement emphasizes follow-up activities. A third option could tie the financial incentive only to the final outcome. This arrangement enables providers to emphasize the steps they feel are important to achieving the objective.

**Identify possible adverse effects of the incentive.** Payments designed to achieve well-defined outcomes sometimes have unintended consequences (5). Because true health care "quality" is difficult to observe, incentives often focus on easily observed metrics like the proportion of patients who receive regular tests or engage in prescribed activities. In addition, people tend to allocate more effort to the activity that is rewarded, resulting in unintended degradation of performance in other areas. In the primary and secondary education sector, pay-for-performance plans have become popular. These plans pay teachers and administrators for improving their students' scores on standardized tests. Such incentives can be effective, but in many instances they have created perverse and unproductive behaviors such as "teaching to the test," manipulating test results, encouraging poorly performing students to not take the test, or reclassifying students to artificially increase performance indicators. In most cases, these responses to financial incentives can be traced to faulty designs in the incentive arrangement or faulty measurements of performance (6).

**Evaluate and report changes in the behavior or outcome in response to the incentive.** Monitoring the results of any incentive arrangement is necessary for its long-term success. In addition to reporting outcomes, other possible effects of the incentive should be studied.

## Developing Incentives

A wide range of financial incentives is applicable to population health, each with advantages and disadvantages (7).

### Flat payments for documented behavior

In this arrangement, decision makers receive a fixed payment for attaining a target or undertaking an action. Such incentives are simple to describe and administer and are widely used in various policy areas. For example, unrestricted cash payments to low-income families for choices that increase human capital and break the cycle of poverty are being tested in several sites. Such incentives are known as conditional cash transfer programs.

A privately funded New York City program called Opportunity NYC offers cash payments to parents if they document particular actions designed to increase the school attendance of their children, improve their children's academic achievement, and increase their preventive health visits (eg, documented prenatal care for mothers and health care for young children) (3). The payments are substantial and together can raise family income by an estimated 25% to 30% (approximately $4,000 to $6,000 annually). Nonprofit partners pay, for example, $25 for attending parent-teacher conferences, $100 for a preventive health screening, and $150 per month for maintaining full-time employment. A similar program, Mexico's Oportunidades, has demonstrated increases in the educational and health outcomes of its participants, including significant increases in school attendance, achievement, and preventive health visits (8).

An advantage of such a plan is that it induces initial action that otherwise may not have been undertaken. However, such a flat payment does not reward continuity of effort after the goal has been achieved. Another disadvantage is that decision makers (in this case, parents) may be paid for choices they would have made anyway. Such payments are "windfalls" to the decision maker and lead to unproductive increases in costs to the payer.

### Graduated payments for documented behavior

A variation of the flat-payment arrangement is a schedule that increases payments as documented behavior moves toward the goal. For example, states operate child support enforcement programs with a mix of federal and state funds. The federal government matches every $1 a state spends on child support enforcement with $2 of federal funds. The federal government also offers graduated incentive payments to states as they achieve better performance on specified indicators (eg, the percentage of cases with paternity established or with on-time payments). Most analyses conclude that these incentive arrangements have been effective in increasing total child support collection nationally (9). The advantage of such graduated plans is that they maintain and increase the incentive for sustained efforts toward attainment of the objective.

### Financial penalties

Penalizing behaviors that do not meet goals is a common form of financial incentive, especially for environmental targets. The primary example is the "effluent charges" policy that has long been advocated by economists. In 1 variant of this proposal, a target level of emissions (eg, carbon dioxide) would be specified for organizations that discharge the gas. If they do not meet this target, they would be required to pay a fee for each unit of discharge beyond the target level.

Penalty arrangements also might be appropriate for some health care targets. For example, a meaningful target might be that 80% of a primary care physician's patients have blood pressure lower than 140/90 mm Hg. A penalty of $200 could be imposed for every percentage point that a provider's patient base falls short of the target. If only 75% of the patient base has normal blood pressure after a predetermined length of time, the penalty would be $1,000. This negative incentive could also be graduated in accordance with the extent to which behavior falls below expectations. Although some adjustment for risk is essential in such an arrangement, the difficulties of specifying an appropriate adjustment must be recognized.

Imposing penalties for inadequate attainment is like imposing a fine; it signals poor performance. Such a signal could lead to provider resentment, discouragement, erosion of loyalty, and opposition to other incentives. From the organization's point of view, imposing penalties avoids a monetary payment, whereas offering incentives does not. Finally, such penalty arrangements could encourage providers to discourage or reject high-risk patients, who would then have to seek alternative care arrangements, potentially resulting in no care or inferior care.

### Payment systems for bundled services

Incentive payment systems may be structured to allow the decision maker discretion over the bundle of procedures and processes chosen to attain an objective. Such a bundled incentive focuses the incentive payment only on the overall health outcomes at issue, rather than each of the actions or behaviors that lead to them.

Bundled payment arrangements are common in the private sector, and are often known as fixed-price contracts. For example, a municipality may contract with a private construction company to resurface a road but stipulate only the required characteristics of the resurfaced road, allowing the construction company wide discretion in choosing the best production process to accomplish the resurfacing.

In health care, prospective payment systems provide a single comprehensive payment for an episode of care, on the basis of the diagnosis. In the context of Medicare reimbursement for hospital stays, each patient is classified into a diagnosis-related group (DRG) and the hospital is paid a flat rate for the DRG (after adjusting for outliers or early release), regardless of the actual services provided. The motivation for this financial incentive system is to establish a base payment for providing a typical set of services, thereby eliminating the incentive for providers to charge more for profitable — though unproductive and discretionary — follow-up services or secondary diagnoses. The system lowers costs by reducing lengths of stay, reducing intensity of care, or improving efficiency of hospital operations. However, these incentives may cause providers to manipulate the demand for services, for example, by disaggregating hospital stays into multiple admissions or, in the provision of primary care services, attempting to attract healthy patients.

Moreover, this sort of incentive arrangement can lead to "risk shifting"; for example, by paying a group-specific fixed amount, the payer shifts the risk of variable treatment costs to the health care provider. This shift may encourage excessively restrictive (and thereby inefficient) care than a DRG typically warrants or the movement of patients into an inappropriate DRG.

### Nonfinancial incentives

Nonfinancial inducements to enhanced performance are common. In the private sector, a typical scheme might provide additional paid vacation days to high-performing workers or public recognition such as employee of the month. In the education sector, schools might try to attract teachers and improve their performance by streamlining hiring practices, offering comprehensive mentoring, reducing class sizes, and providing strong administrative support. In selected settings, these incentives can be effective (10).

Nonfinancial incentives may also work in the health care sector. Although people are often constrained in their health care choices, information on the cost and quality of providers could result in a reallocation of demand and revenue toward providers with the best results. If such information were mandated and widely used, hospitals and providers might be pressured to improve their performance in the dimensions indicated (11). Comparative effectiveness research has been proposed to evaluate the benefits, risks, and costs of treatment options. To affect medical treatment and reduce health care costs, the results of comparative effectiveness analyses would have to be not only persuasive but also used in ways that change the behavior of providers and patients (12,13).

### Conclusion

Designing good incentive programs is difficult. By focusing rewards on choices that promote health outcomes, quality improvements, and efficiency gains, health care organizations and their patients appear to have much to gain. However, some incentives may foster undesirable competition, may become subjective or political, or may be poorly aligned with the collegial norms of the organization. Evidence for the benefits of incentive programs has been slow to emerge, partly because reliable assessment of incentive arrangements requires detailed research about how behaviors have changed because of the incentives. Nevertheless, the potential for such programs seems large; comparative effectiveness research should be considered for both financial and nonfinancial incentives. Additional research is necessary to support the effective design of incentive programs and to assess them comprehensively.
